# Effect of C_1_ Single‐door Laminoplasty on Symptomatic Atlas Canal Stenosis

**DOI:** 10.1111/os.13352

**Published:** 2022-08-26

**Authors:** Linwei Chen, Xiuliang Zhu, Bin He, Qixin Chen, Fangcai Li

**Affiliations:** ^1^ Department of Orthopedic Surgery, the Second Affiliated Hospital Zhejiang University School of Medicine Hangzhou Zhejiang Province China; ^2^ Orthopedics Research Institute of Zhejiang University Hangzhou Zhejiang Province China; ^3^ Key Laboratory of Motor System Disease Research and Precision Therapy of Zhejiang Province Hangzhou City Zhejiang Province China; ^4^ Clinical Research Center of Motor System Disease of Zhejiang Province Hangzhou Zhejiang Province China; ^5^ Department of Radiology, Second Affiliated Hospital Zhejiang University School of Medicine Hangzhou City Zhejiang Province China

**Keywords:** Atlantoaxial fusion, Hypoplasia of atlas, Single‐door laminoplasty, Symptomatic atlas canal stenosis

## Abstract

**Objective:**

To verify the effect of single‐door laminoplasty combined with atlantoaxial fusion in the treatment of symptomatic atlas canal stenosis.

**Methods:**

This is a single‐center retrospective analysis. From February 2014 to January 2019, 16 patients (five were females) with an average age of 63.4 years (56–71 years) were enrolled in this study. Patients with compressive cervical myelopathy with CT scan showed an inner sagittal diameter (ISD) of C_1_ less than 29 mm or C1 canal space available for cord (SAC) of <12 mm were included, while isolated C1 stenosis without myelopathy or isolated C1 stenosis without atlantoaxial subluxation were excluded in this study. All patients underwent continuous heavy‐weight skull traction, atlas single‐door laminoplasty and atlantoaxial fusion. The differences in the pre‐ and post‐operative inner sagittal diameter, space available for cord, atlas‐dens interval (ADI) and compression of the spinal cord were analyzed by using CT and MRI. Functional evaluation was performed by using the Japanese Orthopaedic Association scoring system and the Neck Disability Index scoring system.

**Results:**

Single‐door laminoplasty provided a full decompression for the spinal cord while retaining the whole posterior arch. No complications were encountered except a superficial wound infection in one patient. At final follow‐up, The ADI was significantly reduced from 5.2 ± 1.8 mm to 1.7 ± 0.6 mm after surgery on average (*P* < 0.05). Average inner sagittal diameter of C1 was increased from 26.3 ± 2.6 mm to 34.9 ± 2.9 mm and the space available for cord was increased from 6 ± 1.7 mm to 17.8 ± 3.6 mm (*P* < 0.05). Meanwhile, the Japanese Orthopaedic Association (JOA) score of the 16 cases was improved from 11.4 ± 1.8 to 14.1 ± 1.4 on average (*P* < 0.05). The postoperative neck pain VAS score decreased significantly, from 2.6 ± 1.0 preoperatively to 1.3 ± 0.9 postoperatively (*P* < 0.05). The influence of neck pain on patient's life was improved from 17.8 ± 3.9 to 13.9 ± 3.3 after surgery (*P* < 0.05). At the last follow‐up, the healing of the hinge fracture and the fusion between atlas and axis were observed in all patients.

**Conclusions:**

Single‐door laminoplasty combined with atlantoaxial fusion not only provides enough space for decompression but also offers intact arch for bone grafting, suggesting that it might provide a more feasible method for the correction of symptomatic atlas canal stenosis.

## Graphical abstract

- Single‐door laminoplasty combined with atlantoaxial fusion is a safe and simple treatment for symptomatic atlas canal stenosis.
- Atlas hypoplasia usually developed symptomatic myelopathy due to degenerative factors, mainly atlantoaxial subluxation. Tha is why atlantoaxial fixation and fusion was recommended.
- C1 lamintectomy is a traditional and useful treatment for atlas hypoplasia, however, this operation may make the instability between C1‐2 worse.
- Single‐door laminoplasty not only provides enough space for decompression but also retains C1 arch for bone grafting.
- At the last follow‐up, the healing of the hinge fracture and the fusion between atlas and axis were observed in all patients.

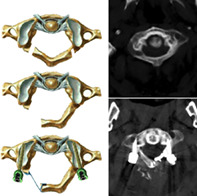

## Introduction

In normal adult individuals, the spinal canal diameter was largest at C1, narrowed from C2 to C5, and slightly widened at C6 and C7, the C4 level was the most stenotic.^1^ Therefore, symptomatic cervical spinal canal stenosis tended to be present more at the subaxial level than at the C1‐2 level. Congenital upper cervical stenosis, especially of the atlas, is quite rare. Hypoplasia of the atlas is defined as an inner sagittal diameter of C1 of 26 mm or less.^2^ Hypoplasia of the posterior arch of the atlas can be divided into two types: a congenital type with partial atlas agenesis caused by spondyloepiphyseal dysplasia congenita and an idiopathic type characterized by a hypoplastic, but complete ring. In cases showing partial atlas defect, symptomatic myelopathy frequently developed at young adults due to anterior movement of the bony remnant causing repeated direct spinal cord trauma with neck extension.^3^ In contrast, people with a complete and small size of atlas alone may not develop symptomatic myelopathy, or developed symptomatic myelopathy at the age of more than 60 years, which suggests that the determining factor for C1 stenosis could be degenerative rather than congenital. According to the literature and our experiences, several pathologies including atlantoaxial subluxation (AAS), ossification of the transverse ligament, retro‐odontoid pseudotumor, basilar invagination and Os odontoideum may have contributed to the development of myelopathy.^4^, ^5^, ^6^, ^7^ Clinically significant myelopathy happened when the canal was further narrowed by dislocating dens, scar encroachment and malformed odontoid process. Congenital small atlas combined with degenerative factors leads to symptomatic myelopathy. The “two‐hit” pathophysiology at the subaxial level can also be applicable to the C1 level.^8^

Symptomatic atlas canal stenosis (SACS) should be treated by operative intervention to prevent neurologic deficit progression. Most patients with SACS require stabilization as well as decompression. Combining a C1 lateral mass screw and C2 pedicle screw could achieve a firm fixation. However, *in situ* fusion without decompression cannot prevent neurologic deficit progression. C1 laminectomy *via* the posterior approach provides enough space for spinal cord in the treatment of SACS, however, this operation may make the instability between C1‐2 worse.^8^, ^9^ To address these problems, C1 laminectomy combined with C1‐C2 fixation can be considered to achieve successful decompression as well as stabilization, however, if sufficient space for bone fusion is not secured following laminectomy, the internal fixation will finally fail. As another way to achieve enough space for the bone graft, the fusion area can be extended to the occipital bone but this may sacrifice the motion of craniocervical junction.

A surgical procedure that decompresses the spinal cord while retaining the C1 arch is needed for C1–C2 stability and cord compression. Laminoplasty has been widely utilized as a surgical procedure of decompression. It is mostly performed from C3 to C7 and rarely at the C1–C2 level. C1 laminoplasty using an allograft spacer could be a useful decompression technique. Application of C1 laminoplasty by using hydroxyapatite spacer without plate augmentation was reported in a case of retro‐odontoid pseudotumor regression.^7^ However, the artificial bone may break. Recently, C1 double‐door laminoplasty combined with atlantoaxial fusion as a new technique was applied in SACS.^10^ By leaving the C1 laminae *in situ*, sufficient space for C1‐2 fusion is secured following laminoplasty. Meanwhile, this technique offered full decompression and sufficient stability. However, since the C1 posterior arch is very small and fragile, double‐door laminoplasty has disadvantages of complicated operation and it is easy to cause secondary fracture. To simplify the operation, we developed single‐door C1 laminoplasty using a titanium miniplate combined with C1–C2 fusion to address this issue. In addition to fusion, decompression was achieved, thereby resolving both canal stenosis and instability. Furthermore, C1 laminoplasty provides sufficient space for C1‐2 fusion. The purpose of this study was to present the details of this surgical technique; and evaluate the clinical and radiographic outcome of C1 single‐door laminoplasty combined with atlantoaxial fusion in patients with SACS. To achieve these purposes, preoperative and intraoperative data of the patients were collected. Radiological and functional evaluation were compared with each other preoperatively, postoperatively, and at the follow‐up.

## Materials and Methods

### Inclusion Criteria and Exclusion Criteria

The characteristics of the patients were as follows: (i) patients experienced posterior neck pain, unstable gait, sensory deficit and positive pathologic signs; (ii) the CT scan showed an inner sagittal diameter (ISD) of C1 less than 29 mm or C1 canal space available for cord (SAC) of <12 mm;^2^ and (iii) The MRI showed spinal cord compression at C1 level.

The exclusion criteria included: (i) isolated C1 stenosis without myelopathy; (ii) isolated C1 stenosis without AAS; (iii) no hypoplasia of atlas on CT or MRI; and (iv) occipitalization of atlas on CT scan.

### General Data

Finally, we retrospectively reviewed 16 cases of SACS between February 2014 and January 2019. All patients experienced posterior neck pain, unstable gait, sensory deficit and positive pathologic signs. X‐ray images in the flexion‐extension view and sagittal CT reconstruction images were further evaluated for the presence of AAS, basilar invagination and Os odontoideum. AAS was defined as atlas‐dens interval (ADI) >3 mm at flexion. Basilar invagination was confirmed as odontoid process exceed the Chamberlain line by >3 mm. (Table 1).

**TABLE 1:** Characteristics of the patients with C1 stenosis

Case	Age/Gender	Symptom	Combined factors	Treatment	Follow‐up (M)
1	66/M	myelopathy	AAS, OS	CLP + AAF	24
2	66/F	myelopathy	AAS	CLP + AAF	22
3	69/M	myelopathy	AAS, SCS	CLP + AAF + laminoplasty	25
4	67/M	myelopathy	OOF	CLP + AAF	25
5	57/M	myelopathy	AAS, BI	CLP + AAF	24
6	63/F	myelopathy	AAS, BI	CLP + AAF	23
7	67/M	myelopathy	AAS	CLP + AAF	22
8	64/F	myelopathy	AAS	CLP + AAF	24
9	71/M	myelopathy	AAS	CLP + AAF	26
10	66/M	myelopathy	AAS	CLP + AAF	25
11	58/M	myelopathy	AAS, SCS	CLP + AAF + laminoplasty	26
12	63/F	myelopathy	AAS, OS	CLP + AAF	23
13	56/M	myelopathy	AAS, BI	CLP + AAF	27
14	61/F	myelopathy	AAS	CLP + AAF	24
15	57/M	myelopathy	AAS	CLP + AAF	25
16	63/M	myelopathy	AAS, OS	CLP + AAF	26

Abbreviations: AAF, atlantoaxial fusion; AAS, atlantoaxial subluxation; BI, basilar invagination; CLP, C1 single‐door laminoplasty; OOF, Old odontoid fracture; OS, os odontoideum; SCS, subaxial cervical spinal stenosis.

### Surgical Technique

#### Anesthesia and Position

All patients were treated with general anesthesia, placed in the prone position and treated with continuous skull traction with 8–10 kg weight. A preliminary reduction of AAS and basilar invagination could be achieved under C‐arm guided image monitoring.

#### Approach and Exposure

An approximately 8cm midline posterior incision was performed to expose the posterior lamina of C1, C2 and skull. Care should be taken to protect the occipital major nerve and vertebral artery.

#### Pedicle Screw Placement and AAS Reduction

Screws (ø 4.5 mm × 26 mm) (Sanyou, Shanghai, China) were introduced into the C1 lateral mass and screws (ø 4.5 mm × 28 mm) were introduced into C2 pedicles, respectively. To fully reduce the AAS, the C2 screws were used as an anchor to pull C1 posteriorly by tightening the screw head over the rods with preformed curve until ADI of <3 mm was achieved. Mobile C‐arm fluoroscopy was used to confirm the reduction of AAS.

#### C1 Single‐door Laminoplasty and Miniplate Fixation

For C1, single‐door laminoplasty was made at the junctional zone of the lamina and lateral mass by using a high‐speed drill burr. After carefully grinding up until the inner cortex of the lamina, a hinge was formed at the right side of the lamina. The left gutter of the C1 posterior arch was deepened continuously and gently until both the outer and inner cortex of the lamina were all broken through. By opening the left gutter of the C1 posterior arch gently toward the right side, a greenstick fracture was formed at the right side of the lamina, and a door of at least 6 mm width was kept open at the left side of the lamina. A titanium miniplate was clipped into the door at the left side of the lamina. Then, the titanium miniplate was firmly fixed on left side of the lamina with four cortical screws (ø 1.5 mm × 4 mm).

#### Autogenous Iliac Bone Graft

**Fig. 1:**
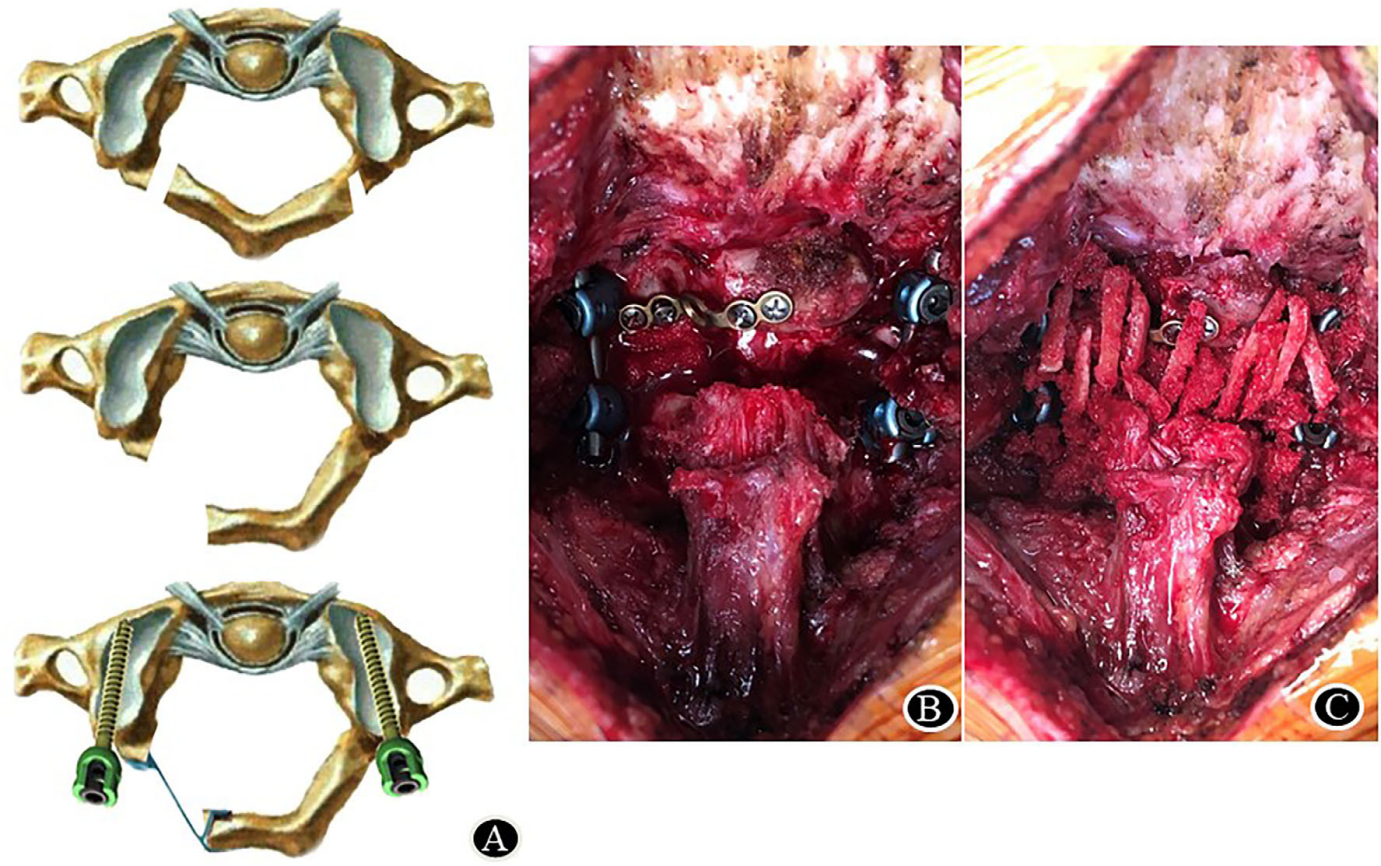
Intra‐operative image for C1 single‐door laminoplasty and AAF. (A), Schematic diagrams of C1 laminoplasty; (B), Intraoperative view showing a successful laminoplasty in atlas; (C), Decortication of the lamina of C1 and C2 was prepared and autogenous bone chips harvested from iliac crest was grafted for fusion

Decortication of the lamina of C1 and C2 was prepared by using a high‐speed drill burr, autogenous bone granules harvested from iliac crest were grafted for fusion (Fig. 1). Autogenous bone granules were grafted on the hinge side and a piece of gelatin sponge was placed in the door at the contralateral side to ensure that the posterior implanted bone shavings do not enter the spinal canal, resulting in compression of the spinal cord.

#### Postoperative Protocol

Partial weightbearing with neck brace immobilization was allowed immediately after surgery and full weightbearing was allowed at 3 months postoperatively.

### Radiological Evaluation

Cervical spine X‐ray, CT and MRIs were obtained from each patient before surgery, after surgery and at the last follow‐up. Radiographic evaluation was performed by two independent radiologists using the PACS system. Radiographic parameters including ISD (inner sagittal diameter of C1), SAC (space available for spinal cord at C1 level) and ADI (atlas‐dens interval) were all measured on CT scan. To evaluate the effect of spinal cord decompression, midsagittal T2‐weighted MRI images were obtained to observe the subarachnoid space and spinal cord.

### Functional Evaluation

The neurological status of the patients was evaluated using Japanese Orthopaedic Association (JOA) scoring system preoperatively and 2 years after operation.^11^ To assess the impact of neck pain on daily activities, the visual analogue scale (VAS) and neck disability index (NDI) score were evaluated by an independent surgeon preoperatively and 2 years later.^12^

### Statistical Analysis

The average ISD, SAC, ADI, JOA score, VAS score and NDI score were expressed as means (±SD). A paired Student's *t*‐test was used for comparisons of continuous data. Statistical analysis was performed with SPSS 18.0 software for Windows (SPSS, Chicago, IL, USA). Differences at a level of *P* < 0.05 were considered statistically significant.

## Results

### General Characteristics

**Fig. 2:**
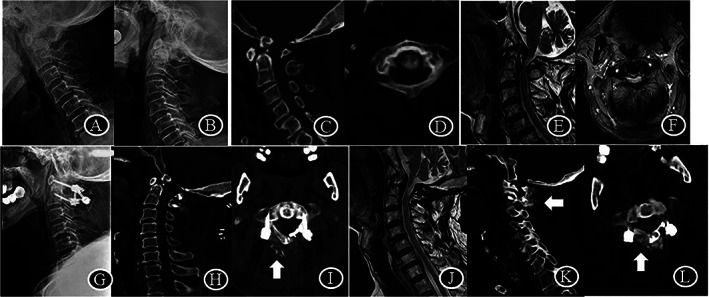
Radiological data of patient no. 1 with atlas hypoplasia and os odontoideum. (A–D), ISD of 23 mm, SAC of 3 mm and ADI of 4 mm pre‐operation; (E, F), T2–weighted images show constriction of the dural sac at the level of the atlas; (G–I). ISD of 30 mm, SAC of 20 mm and ADI of 1 mm immediately post‐operation. Note the autogenous iliac bone graft; (J). 24 months later after surgery, MRI shows subarachnoid space around the dural sac at the level of the atlas; (K, L). The reduction and decompression maintained at the last follow‐up. The healing of the hinge fracture and the fusion between C1 posterior arch and C2 lamina was identified

**Fig. 3:**
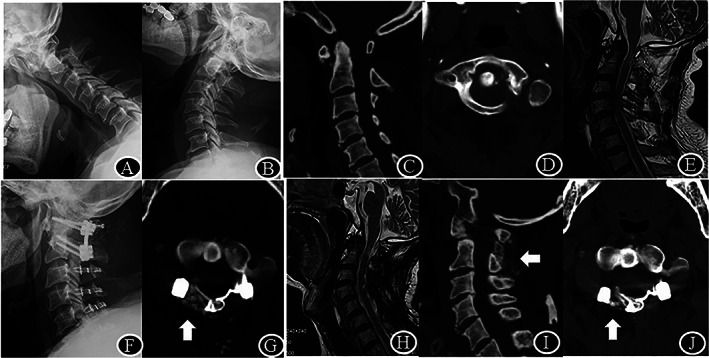
The imaging of patient no. 3, male, diagnosed as atlas hypoplasia and subaxial cervical spinal stenosis. (A–D), ISD of 29 mm, SAC of 8 mm and ADI of 4 mm pre‐operation; (E), Preoperation MRI shows posterior compression at the level of atlas and C3/4, C4/5; (F), Postoperative X‐ray of the same patient shows C1‐2 pedicle screws and C1, C3‐6 laminar mini‐plate; (G), ISD of 36 mm, SAC of 23 mm and ADI of 1.5 mm on axial‐CT immediately post‐operation and autogenous iliac bone graft; (H), Sagittal T2–weighted image shows full posterior decompression at 25 months after laminoplasty; (I, J), Sagittal and axial CT identified the healing of the hinge fracture and successful interlaminar fusion

**Fig. 4:**
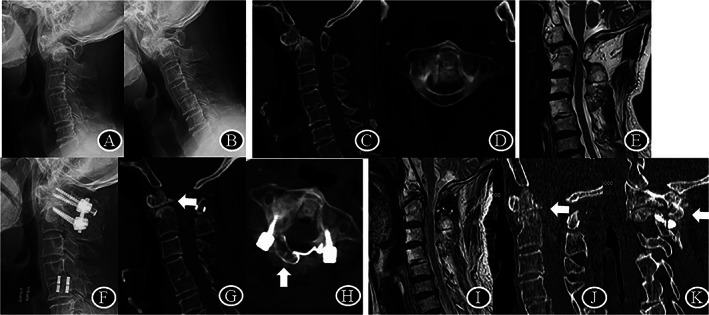
The imaging of patient no. 4, male, who suffered from an old and displaced odontoid fracture. (A–D), Preoperation SAC of 5 mm; (E), Preoperative midsagittal T2‐weighted MRI demonstrating the spinal cord was compressed by the dens and posterior ring of C1; (F–H), Postoperative CT scan, demonstrating full reduction of dens fracture and SAC of 12 mm immediately post‐operation; (I), Postoperative T2‐weighted MRI, demonstrating full decompression at 25 months after laminoplasty; (J, K), Sagittal CT scan, demonstrating successful dens fracture healing and interlaminar fusion between C1‐C2

All patients were followed an average of 24.9 (range, 23 to 28) months. Except atlas hypoplasia, degenerative factors including AAS, Os odontoideum, basilar invagination, subaxial cervical spinal stenosis and old odontoid fracture were identified in 16 patients (Figs 2, 3, 4). Five cases were females and 11 cases were males, with an average age of 63.4 years. Fourteen patients underwent C1 laminoplasty and C1‐2 fusion, while two patients had both atlas hypoplasia and subaxial cervical spinal stenosis underwent both C1 and C3‐7/3–6 single‐door laminoplasty (Table 1, Fig. 3). The average operation time was 132 ± 15 min and the average blood loss was 104 ± 9 mL.

### Intraoperative Findings

(i) The lamina at the junctional zone of the lamina and lateral mass of C1 is thicker than C3‐6. Care should be taken in dealing with the inner cortex of the lamina; (ii) laminoplasty should be completed before introducing the C1 lateral mass screws, but the screw trajectory must be prepared before laminoplasty; and (iii) stump of at least 6 mm width should be remained at the left side of the lamina to allow the fixation of a titanium miniplate.

### Radiological Outcomes

By heavy weight skull traction, the ADI was significantly reduced from 5.2 ± 1.8 mm before surgery to 1.7 ± 0.6 mm after surgery on average (*P* < 0.0001). Meanwhile, enlargement of C1 canal *via* posterior approach was achieved in all cases. At final follow‐up, ISD was increased from 26.3 ± 2.6 mm to 34.9 ± 2.9 mm, there was 8.8 mm increase in sagittal diameter compared to that before surgery (*P* < 0.0001). Meanwhile, SAC was increased from 6 ± 1.7 mm to 17.8 ± 3.6 mm, there was 11.8 mm correction in stenosis compared to that before surgery (*P* < 0.0001) (Table 2). At the final follow‐up, the healing of the hinge fracture and the fusion between C1 posterior arch and C2 lamina were observed in all patients (Figs 2, 3, 4).

**TABLE 2:** Comparison of radiological results before and after the operation

Case	ISD (mm)		SAC (mm)		ADI (mm)	
	Pre	Post	Pre	Post	Pre	Post
1	23	30	3	20	4	1
2	27	34	5	18	8	2
3	29	36	8	23	4	1.5
4	32	41	5	12	1	1
5	27	37	7	15	5	2
6	26	36	5	16	4	1.5
7	27	38	4	18	6	2
8	24	32	8	25	7	2
9	22	30	6	14	8	1.5
10	26	33	5	16	5	1
11	24	37	8	15	6	1
12	24	34	6	17	4	2
13	28	35	9	24	4	3
14	25	34	4	18	4	2
15	29	38	7	16	6	1
16	27	33	6	17	7	2
Mean	26.3 ± 2.6	34.9 ± 2.9	6 ± 1.7	17.8 ± 3.6	5.2 ± 1.8	1.7 ± 0.6
*P*	<0.0001		<0.0001		<0.0001	

Abbreviations: ADI, atlas‐dens interval; ISD, inner sagittal diameter; SAC, space available for cord.

### Clinical Outcomes

No intraoperative or postoperative complications were encountered except a superficial wound infection in one patient that was treated by sensitive antibiotics. At the final follow‐up, each patient exhibited improvement of neurological symptoms. The JOA score of the 16 cases was improved from 11.4 ± 1.8 to 14.1 ± 1.4 (*P* < 0.0001). The VAS score was decreased from 2.6 ± 1.0 to 1.3 ± 0.9 (*P* < 0.005). The influence of neck pain on patient's life was improved from 17.8 ± 3.9 to 13.9 ± 3.3 after surgery (*P* < 0.005) (Table 3).

**TABLE 3:** Comparison of functional results before and after the operation

Case	JOA score		NDI score		VAS score	
	Pre	Post	Pre	Post	Pre	Post
1	13	15	16	15	2	1
2	9	13	20	18	3	1
3	10	13	18	16	2	3
4	12	16	10	10	5	2
5	10	15	14	10	3	0
6	12	15	22	12	3	1
7	12	14	24	20	2	2
8	13	13	22	15	2	2
9	14	15	20	15	3	2
10	13	16	21	18	4	0
11	9	13	13	9	3	0
12	13	14	22	13	2	1
13	12	16	15	14	1	1
14	10	12	18	9	1	0
15	8	12	15	13	3	2
16	12	14	15	15	2	2
Mean	11.4 ± 1.8	14.1 ± 1.4	17.8 ± 3.9	13.9 ± 3.3	2.6 ± 1.0	1.3 ± 0.9
*p*	<0.0001		<0.005		<0.005	

Abbreviations: JOA Score, Japanese Orthopaedic Association Score; NDI Score, Neck Disability Index Score; VAS Score, Visual Analogue Scale.

## Discussion

SACS is a rare disease that may challenge every spine surgeon.^2^, ^4^, ^5^, ^13^, ^14^, ^15^, ^16^ This study introduced reduction of AAS, single‐door laminoplasty of atlas and atlantoaxial fusion in the treatment of SACS. We found this new method could not only completely reduce AAS but also fully enlarge the inner sagittal diameter of atlas, and achieve C1‐2 fusion satisfactorily.

### Etiology and Diagnosis of SACS

The atlas originates from three ossification centers that arise from the rostral portion of the first sclerotome. Hypoplasia of the atlas could be the result of premature fusion of the posterior ossification centers.^17^ Musha and Mizutani measured 300 adult Japanese and defined hypoplasia as 29.4 mm in females and 30.5 mm in males.^9^ Kelly *et al*. measured 543 vertebral specimens and defined an ISD of 26 mm or less as hypoplasia of the atlas.^2^ Yamahata *et al*. reported 13 cases of SACS with average C1 ISD and SAC of 26.9 ± 2.4 mm and 12.8 ± 4.1 mm, respectively.^8^ In our case series, the average ISD and SAC measured on helical CTs was approximately 26.3 ± 2.6 mm and 6 ± 1.7 mm, respectively. One 67 year old man with an old odontoid fracture had an ISD of 32 mm, which could not be diagnosed as a narrow ring. We still included this patient in this research because the SAC in this patient was only 5 mm. Besides the dislocation of old fracture, massive callus and scar invaded into the spinal canal, making the spinal cord compression worse.

### Treatment Strategy and Advantages of C1 Laminoplasty

The prevailing treatments for SACS include indirect and direct decompression. The indirect decompression consists of AAS reduction and atlantoaxial fusion. Continuous heavy‐weight skull traction helps reducing the upward and backward displacement of the odontoid process. However, this reduction may not be sufficient in cases with a narrow atlas or a rigid AAS. Direct decompression may be more efficient in expanding SAC by removing either dislocating process or posterior arch. Direct decompression *via* anterior approach challenges most spinal surgeons, which needs much higher skills and experiences in anatomy and decompression. Decompression *via* posterior approach may diminish learning curve because most spine surgeons are familiar with this approach. C1 laminectomy is the traditional and prevailing method in treating C1 canal stenosis. Although most patients demonstrated neurological improvement after C1 laminectomy,^18^, ^19^ laminectomy alone was only applicable in cases where AAS is either absent or minor. However, since most SACS simultaneously associated with AAS, decompression alone could make the instability worse.^16^ Furthermore, finite element analysis reveals that stress distribution concentrates in the anterior arch after C1 laminectomy, leading to anterior arch fracture despite no inciting trauma.^20^ The incidence of anterior arch fracture was 14.2% especially in cases with a large inferior facet angle (defined as the coronal inclination angle of the C1/2 facet as measured on CT).^21^ C1‐2 facet joint fusion may make up the deficiency of simple laminectomy, nevertheless, the potential sacrifice of the occipital major nerve and vertebral artery limits its application. Comparatively, the result of our study indicates that C1 laminoplasty combined with C1‐2 interlaminar fusion had considerable advantages over laminectomy with or without facet joint fusion. C1 laminoplasty not only maintained the integrity of C1 ring, thus avoiding anterior arch fracture, but also provided the foundation of bone grafting. Meanwhile, C1‐2 interlaminar fusion could be performed by most orthopedic surgeons because this manipulation is very safe and convenient compared to facet joint fusion.^20^, ^21^ The main advantage of this surgery is that it provides full decompression and strong fusion simultaneously.

### Clinical and Radiographic Outcomes after C1 Laminoplasty and C1‐2 Fusion

In this study, complete decompression and rigid fusion were achieved in all cases. The AAS was fully reduced because ADI was restored from 5.2 ± 1.8 mm to 1.7 ± 0.6 mm postoperatively. The sagittal diameter of C1 was significantly increased from 26.3 ± 2.6 to 34.9 ± 2.9 mm, and the cervical canal at the C1 level was increased from 6 ± 1.7 to 17.8 ± 3.6 mm, there was 11.8 mm correction in stenosis compared to that before surgery. Spinal cord compression was significantly relieved and the JOA score was improved from 11.4 ± 1.8 to 14.1 ± 1.4 on average during the follow‐up. Successful interlaminar fusion between C1 and C2 was observed in all patients. The reconstruction of the spinal stability significantly relieved pain and improved the quality of life at the 24‐month follow‐up.

### Development of C1 Laminoplasty Technique

The most ideal treatment for SACS is sufficient decompression, posterior arch retainment and atlantoaxial fusion. Laminoplasty has been widely utilized as a surgical procedure of subaxial cervical decompression and rarely at the C1–C2 level.^22^ Kim *et al*. first applied double‐door C1 laminoplasty and C1‐2 fusion in the revision surgery of a 66‐year‐old man with SACS.^10^ However, the C1 posterior arch is so small and fragile, especially in Chinese people, that bilateral hinge fracture and internal fixation present a major challenge to surgeons. Our technique is a modification of the double‐door C1 laminoplasty which has several advantages: (i) it allows easier laminoplasty by reducing the possibility of fracture during the screw fixation of the hinge fracture; (ii) it provides more space for bone grafting than double‐door laminoplasty because the whole posterior arch is intact; (iii) it requires no allograft spacer to keep open the French‐door laminoplasty; and (iv) unilateral hinge fracture allows more rapid healing than bilateral hinge fracture. The final radiological and clinical outcome confirmed our hypothesis. The inner diameter of C1 was increased by 50% and the SAC was enlarged by almost 200%. The healing of the hinge fracture and the fusion between the intact C1 posterior arch and C2 lamina were observed in all patients at the last follow‐up. Finally, the recovery of neurologic impairment was satisfying as JOA score was improved significantly at the last follow‐up.

### Limitations and Future Research

The small sample size was the major limitation of the presented study. Moreover, there were no C1 laminectomy patients as a control group. Single‐door laminoplasty combined with C1‐2 interlaminar fusion is a promising technique for SACS. However, further comparative studies need to be performed to evaluate the efficacy and safety of this method.

### Conclusion

In conclusion, we found congenital C1 stenosis and degenerative pathologies both contributed to the development of SACS. Sixteen patients with SACS underwent AAS reduction, C1 single‐door laminoplasty and atlantoaxial fusion. An average follow‐up of 24.9 months confirmed enlargement of the narrow atlas, reliable fusion between C1‐C2 and recovery of neurologic impairment. Our findings recommend single‐door laminoplasty combined with atlantoaxial fusion as a safe and effective treatment for SACS.

## Ethical Approval

This study with human participants was approved by constituted Ethics Committee of the second affiliated hospital of Zhejiang University and conforms to the provisions of the Declaration of Helsinki.

## Informed Consent

Informed consent was obtained from all individual participants included in the study

## Author Contribution

Linwei Chen: collection and assembly of data; data analysis and interpretation; manuscript writing. Xiuliang Zhu: collection and analysis of data; data interpretation, manuscript writing. Bin He: data analysis and interpretation. Qixin Chen: conception and design; final approval of manuscript. Fangcai Li: revision of the manuscript, operation leader, conception and design.
